# Analyzing the effects of free water modeling by deep learning on diffusion MRI structural connectivity estimates in glioma patients

**DOI:** 10.1371/journal.pone.0239475

**Published:** 2020-09-25

**Authors:** Leon Weninger, Chuh-Hyoun Na, Kerstin Jütten, Dorit Merhof

**Affiliations:** 1 Imaging & Computer Vision, RWTH Aachen University, Aachen, Germany; 2 Department of Neurosurgery, University Hospital RWTH Aachen, Aachen, Germany; University of North Carolina at Chapel Hill, UNITED STATES

## Abstract

Diffusion-weighted MRI makes it possible to quantify subvoxel brain microstructure and to reconstruct white matter fiber trajectories with which structural connectomes can be created. However, at the border between cerebrospinal fluid and white matter, or in the presence of edema, the obtained MRI signal originates from both the cerebrospinal fluid as well as from the white matter partial volume. Diffusion tractography can be strongly influenced by these free water partial volume effects. Thus, including a free water model can improve diffusion tractography in glioma patients. Here, we analyze how including a free water model influences structural connectivity estimates in healthy subjects as well as in brain tumor patients. During a clinical study, we acquired diffusion MRI data of 35 glioma patients and 28 age- and sex-matched controls, on which we applied an open-source deep learning based free water model. We performed deterministic as well as probabilistic tractography before and after free water modeling, and utilized the tractograms to create structural connectomes. Finally, we performed a quantitative analysis of the connectivity matrices. In our experiments, the number of tracked diffusion streamlines increased by 13% for high grade glioma patients, 9.25% for low grade glioma, and 7.65% for healthy controls. Intra-subject similarity of hemispheres increased significantly for the patient as well as for the control group, with larger effects observed in the patient group. Furthermore, inter-subject differences in connectivity between brain tumor patients and healthy subjects were reduced when including free water modeling. Our results indicate that free water modeling increases the similarity of connectivity matrices in brain tumor patients, while the observed effects are less pronounced in healthy subjects. As the similarity between brain tumor patients and healthy controls also increased, connectivity changes in brain tumor patients may have been overestimated in studies that did not perform free water modeling.

## 1 Introduction

Structural connectomes are one of the cornerstones of quantifying the human brain and its macro-scale connectivity in-vivo. These connectomes are based on a parcellation of the brain as well as on white matter (WM) diffusion tractography. As this fiber reconstruction is limited due to macroscale imaging, the accuracy, interpretability, and generalizability of structural connectomes created from diffusion MRI (dMRI) is still limited [1]. Recently, it has been shown that WM tractography is also influenced by free water partial volume effects [2], which might be especially relevant in surgical treatment planning of tumor patients with large edema. Several methodological improvements have been proposed to address this issue [3–5].

Including a free water model (FWM) in diffusion tractography aims at correcting two distinct, yet closely related aspects: 1) partial volume effects due to a voxel size larger than brain microstructures, e.g., at the border between the corpus callosum and ventricles, and 2) partial volume effects due to a voxel-wide mixture of parenchyma with free water, which exists in presence of peritumoral edema. Thus, these two artifacts occur at different scales. While an increase in image resolution would reduce the number of type 1 artifacts (clear border between distinct materials), there would be no effect on the number of type 2 artifacts (infiltration).

The majority of studies analyzing the effects of including a FWM focus on scalar-valued maps such as fractional anisotropy (FA) derived from dMRI measurements. Measurable effects were shown for associations with delusions in chronic schizophrenia [6], stress-related neural pathology in depression [7], classification of Parkinson’s disease and atypical parkinsonism [8], target definition in glioblastoma radiotherapy [9], as well as for the diagnosis of mild cognitive impairment and Alzheimer’s disease [10].

However, structural connectomes for multivariate analysis of glioma patients [11, 12] are often implemented without explicitly modeling the free water compartment. Especially in the presence of peritumoral edema, future studies could enhance the accuracy and predictiveness of these multivariate connectomes by including a FWM in diffusion tractography.

A variety of different free water modeling techniques have been published. Loosely, they can be categorized into methods that are applicable to single-shell diffusion data, and methods that can only be applied to multi-shell acquisitions. For multi-shell acquisitions, it is possible to directly fit an appropriate mathematical model to the raw diffusion data in order to estimate the water fraction [13]. Such a model can also be refined to include diffusion kurtosis effects [14]. Different multi-compartment models can also include isotropic compartments, and thus reconstruct fiber directions while taking account of vasogenic edema. Instead of assuming a fixed diffusivity of compartments, in recent publications the isotropic compartments is fitted using a spectrum of diffusivities. Modeling vasogenic edema with agarose gel, those multi-compartment models can be verified in-vitro [15, 16].

However, state-of-the art mathematical models are often not applicable to clinical data. First, they rely on multi-shell data, which is often not available in clinical settings. Second, they require a lot of computing power and take more time than available when working with patients.

For single-shell acquisitions, which are common in clinical settings, a direct model fit is not possible. Instead, some sort of regularization needs to be introduced. This is possible via spatial regularization [17], or via deep learning as in [18].

In our recent publication [19], we showed that a novel deep learning based method, where the neural network is fitted individually for each subject, produces very good results on synthetic data, healthy subjects and brain tumor patients. The method does not depend on a specific MRI protocol, and produces reliable results across a range of acquisition settings. Under https://github.com/weningerleon/DLFreeWaterModel, an open source implementation in python is available together with an exemplary application. With diffusion tractography-based estimation of fiber-connectivity being applied for surgical planning with the aim of maximal safe tumor resection, but preservation of functionally relevant white-matter tracts, we analyzed how this free water mapping and elimination technique influences tractography-based structural connectomes for brain tumor patients.

As it is not possible to obtain a groundtruth connectome for lesioned brains, the changes in connectivity when including the FWM are compared to an age- and gender-matched healthy control group, as well as to the individual contralateral hemisphere. As inter-hemispheric diffusion MRI variance has been shown to be comparable to the inter-subject variance in healthy subjects [20], we assumed that an increase in the inter-hemisphere as well in the inter-subject similarity of the non-lesioned brains would be a first indicator of the plausibility of tractography findings. Comparing tumor patients to the control group, the lesions should cause stronger diversions from the mean connectome. However, the free-water effects in peritumoral edema also cause diversion in the connectivity estimates. We hypothesised that including the FWM should lead to reduced diversions due to free-water effects, while preserving true white-matter alterations due to the lesion itself. Thus, even though different tumor characteristics such as tumor volume, location or growth kinetics have varying impact on the structural connectome, the FWM should lead to a relative increase in inter-subject and inter-hemisphere similarity especially in those patients with marked perifocal edema, while preserving stronger similarities in healthy subjects compared to tumor patients.

## 2 Materials and methods

### 2.1 Data

35 patients with cerebral gliomas (11 WHO IV, 15 WHO III, 9 WHO I/II), as well as 28 age- and sex-matched controls were prospectively enrolled in a study at the University Hospital Aachen.

All subjects underwent T1 and dMRI scans. For the T1 acquisition, the sequence was as follows: TE = 2.01 ms, TR = 2300 ms, 176 slices with a slice thickness of 1 mm, flip angle = 9°, field of view = 256 mm, voxel size = 1 mm isotropic, and a 256 × 256 matrix. The dMRI images were single-shell with b-value = 1000 s/mm^2^, one b = 0 s/mm^2^, TE = 81 ms, TR = 6300 ms, anterior-posterior phase encoded, 64 gradient directions, 55 axial slices, FoV = 216 mm and an isotropic voxel size of 2.4 mm. In addition, one b = 0 s/mm^2^ image with reversed phase-encoding blips (posterior-anterior) was acquired to correct susceptibility induced distortions.

All participants gave written informed consent prior to study enrollment. This study was approved by the local ethics committee (EK 294/15), and conducted in accordance with the standards of Good Clinical Practice and the Declaration of Helsinki. Only patients >18 and <80 years of age and with a Karnofsky index of ≥70 were included. Three patients had previously been treated surgically and two of them had additionally received radiochemotherapy. All other patients were naive to tumor-specific treatment prior to enrollment in the study.

### 2.2 Preprocessing

First, the dMRI acquisitions were corrected for susceptibility-induced correction with the FSL TOPUP toolbox [21] as described in [22], and for eddy currents and motion artifacts with FSL EDDY [23]. The reverse-phase encoded image was wrongly acquired or corrupted in five cases. In these cases, only FSL EDDY was applied. Then, the brain was extracted using FSL BET [24].

The anatomical T1 weighted images were segmented into WM, grey matter (GM), and cerebrospinal fluid (CSF) using FSL FAST [25]. Furthermore, a cortical atlas segmentation was extracted using the default FreeSurfer parcellation pipeline [26] and the Desikan-Killiany atlas.

**Fig 1:**
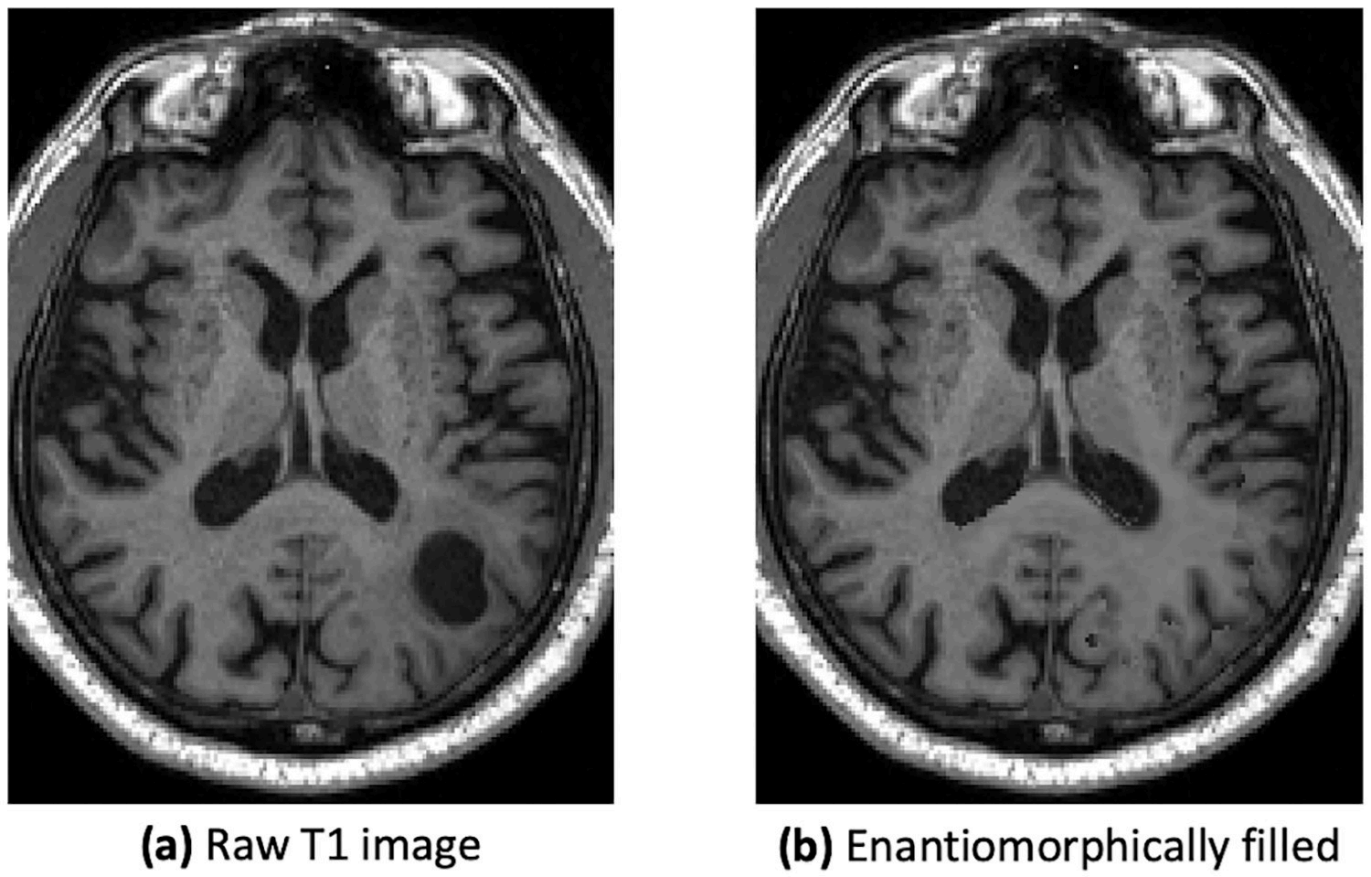
An example of the enantiomorphic filling of the brain tumor. The enantiomorphically filled image was only used for the freesurfer parcellation, as tumors can lead to problems during the fitting process.

However, for brain tumor patients, whole-brain parcellation pipelines often fail to produce reasonable results, as the brain structure can be strongly deformed. To counteract this effect, enantiomorphic filling of the lesion, as proposed by [27], was performed prior to the FreeSurfer parcellation for unilateral gliomas, as visualized in Fig 1.

The obtained FreeSurfer labels were then re-mapped and reduced to lie between 0 and 88, as proposed for connectivity analysis by DIPY [28], the toolbox utilized for tractography and connectivity matrix creation. A list of the final labelled connectome nodes can be found in the appendix (S2 Table).

Finally, the anatomical T1 weighted images were registered to the pre-processed dMRI b0 images using symmetric diffeomorphic image registration as implemented in ANTs [29], and the tissue segmentations and parcellations were transformed into diffusion space.

### 2.3 Free water modeling

The free water modeling technique as presented in [19] was applied to all dMRI acquisitions. It is based on a deep neural network that is trained independently for each subject, with training data extracted from the individual acquisition.

**Fig 2:**
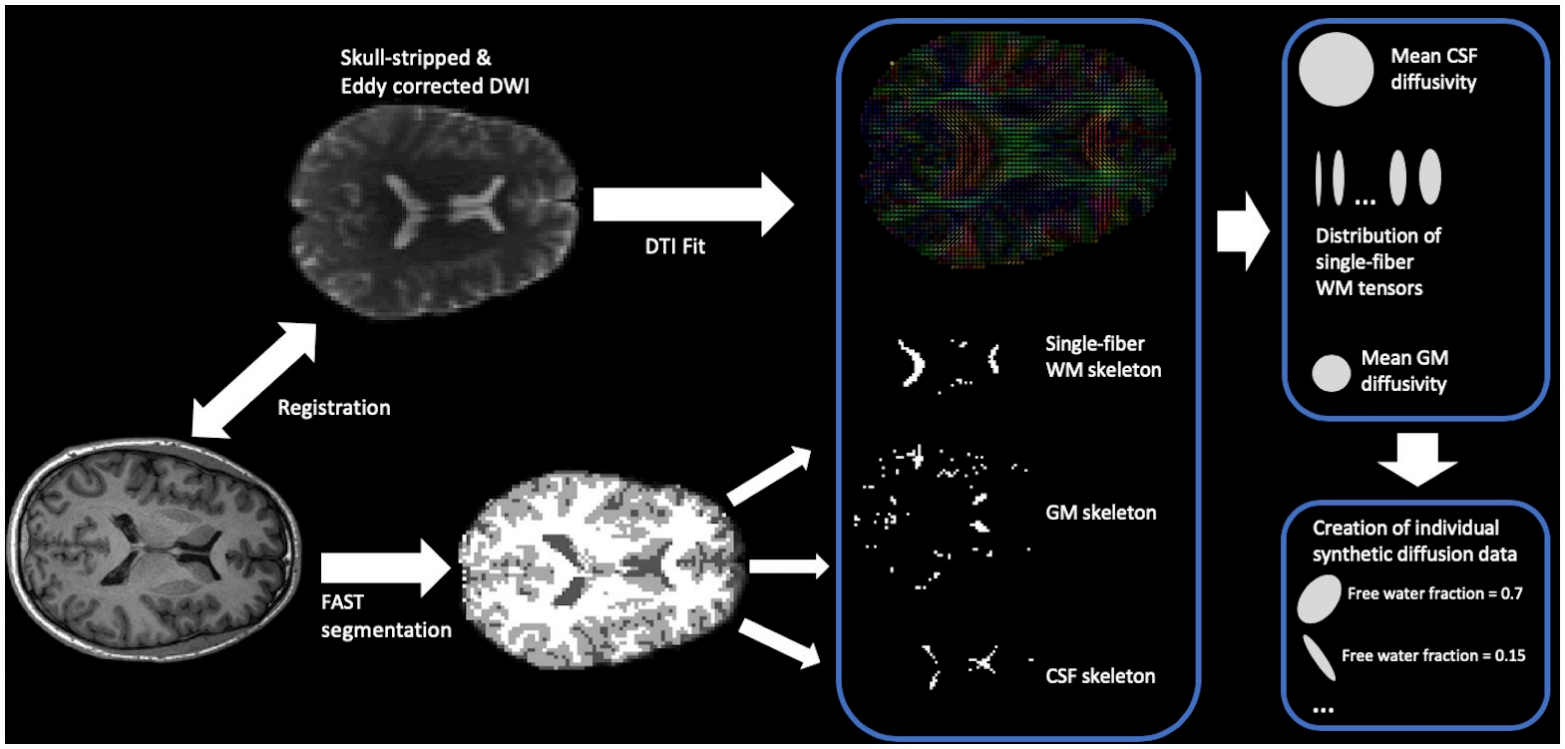
Illustration of the generation of synthetic training data for the individual subject. Gray matter (GM), white matter (WM) and cerebrospinal fluid (CSF) skeletons are extracted from an eroded FAST segmentation map, based on the T1 image. For WM, the map is further reduced to areas where the FA value is higher than 0.7. These tissue maps are then transformed to the diffusion space, where they are used to extract the mean CSF and GM diffusivity, and to sample single-fiber WM voxels. Using the obtained diffusivity values and sample WM voxels, synthetic diffusion data with a known water fraction as well as properties following the characteristics of the individual subject can be created.

First, specific voxels with known tissue properties were located using the co-registered T1 image (Fig 2). Second, by superposition of several voxels with known microstructure, a synthetic dataset that follows individual characteristics, and for which the free water compartment is known, was obtained.

For this procedure, single-fiber WM voxels, GM voxels, as well as CSF voxels were necessary. GM as well as CSF voxels were extracted using the eroded FAST mask. As the diffusivity of these microstructures is isotropic, the mean diffusivity was determined.

Single-fiber WM voxels were extracted from voxels within the corpus callosum with an FA greater than 0.7. This procedure is inspired by the single-fiber response function generation of Constrained Spherical Deconvolution (CSD) [30]. However, contrary to CSD, no mean response function was calculated. Instead, for all suitable WM voxels, the eigenvalues of the diffusion tensors were saved. During creation of the synthetic data set, it was then possible to create single-fiber WM diffusion data by sampling from these sets of eigenvalues.

Using the extracted tissue properties, synthetic diffusion data was created. Single voxels were generated by random superposition of up to three sampled and randomly rotated single-fiber WM voxels, and GM as well as CSF compartments. Finally, Rician noise with an SNR of 20 is used to distort the constructed signal.

The proportions of each sub-compartments—including the proportion of free water—of these synthetic voxels were thus known. As the properties of the single compartments follow the characteristics of the individual brain from which they are extracted, biases due to the MRI-scanner or acquisition settings are circumvented. 250,000 synthetic voxels were created in this fashion per subject, on which a neural network was individually trained to predict the free water fraction. The number of synthetic voxels is thus much higher than the number of nonzero voxels found in a typical diffusion- weighted brain scan in this study, which is about 100,000. Including more voxels also did not lead to a reduced error on the validation set.

For the neural network architecture, a fully connected half-hourglass shape with four fully connected layers was chosen. The shape of single layers in the neural network was automatically determined, based on the number of diffusion weighted acquisition. As input to the neural network, the diffusion signal attenuation was directly used, so the width of the initial layer of the neural network was set to 64, as 64 gradient directions were acquired. The sizes of the hidden layers were determined by halving the size of the previous layer; the last layer finally had only one output, which was regressed against the free water volume fraction with an L2-loss. Thus, the number of artificial neurons of the fully connected layers in this half-hourglass shape was 64-32-16-8-1. Between every two subsequent layers, *tanh* activation functions were used as non-linearities.

For training, the 250,000 created voxels were split into 80% training and 20% validation set. A batch size of 256 and an Adam optimizer with a learning rate of 0.005 was used. On a consumer-grade CPU and an implementation in PyTorch, training of each individual neural network converged after 100 epochs in less than five minutes.

The trained neural network was then used on the signal attenuation of the whole brain to predict the water fraction in all voxels, and the attenuation attributed to the water fraction was subtracted from the original signal. Free water contaminated voxels, i.e., voxels at the border between WM and CSF, or voxels including a vasogenic edema in glioblastoma cases, were corrected.

The hyperparamters employed here were the same as used for previous experiments, where the accuracy of the free water modeling method was evaluated on synthetic data. Those results, as well as a comparison to previously published free water modeling methods on voxel-derived measures such as FA on real data can be found in [19].

### 2.4 Tractography

Two different classes of local tractography methods, deterministic as well as probabilistic tractography, can be utilized for fiber reconstruction [1]. While deterministic methods follow the direction of least hindrance, probabilistic methods estimate the uncertainty in the measurements and create a distribution of possible pathways. At every tracking iteration, the next step is determined by sampling from the fiber orientation distribution function (ODF), which represents the possible dispersion of fiber bundles for each voxel. For both schemes, a large variety of different tractography methods exist, and it is disputed which is better suited for connectome construction. Thus, representative methods of both, deterministic as well as probabilistic tractography, were chosen in this work in order to analyze the effects of the FWM on structural brain connectivity analysis.

For deterministic tractography, the EuDX [31] tracking algorithm was chosen in combination with the Constant Solid Angle diffusion reconstruction model [32]. Both are implemented in DIPY [28]. From the Constant Solid Angle diffusion model, the peaks of the ODF were extracted and served as an estimate for the possible orientations of tract segments. Based on these peaks, EuDX is able to handle crossing fibers.

In the employed probabilistic tractography, the fiber reconstruction was based on CSD [30]. CSD directly computes a fiber ODF, and is thus optimally suited for probabilistic tracking. From the ODF, the next tracking step was randomly sampled using a maximum angle of 30° between current and next step.

Tracking was terminated in both cases based on anatomical stopping criteria, as proposed by [33]. Valid stopping regions were all extracted and parcellated GM regions as described in Section 2.2. CSF and areas outside the brain were invalid stopping areas. Valid white matter for tractography seeding and tracking was defined as areas with FA>0.15. Two different seeding techniques were used, whole WM seeding and WM-GM boundary seeding. The WM-GM boundary was created by taking the intersection of the WM region with the GM area dilated by one voxel in every direction. For every valid seeding voxel, 27 streamline start points were generated. The streamline step size was set to 0.5 mm. Streamlines of less than 1 mm were discarded.

### 2.5 Connectivity matrix creation

**Fig 3:**
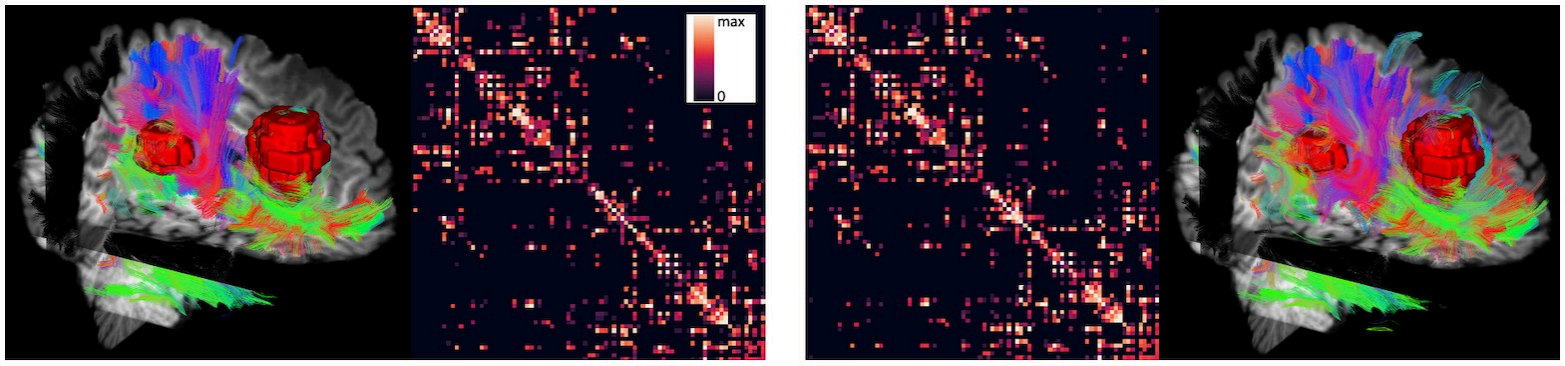
Tractography result and fiber-count connectivity matrix with (right) and without (left) a free water model for a brain tumor patient with a large edema. Here, gray matter interface seeding was used together with deterministic tractography. For better visualization, only every 4th streamline is plotted. The tumor core is visualized in red, and displayed together with three T1 slices in x,y, and z direction as well as streamlines generated from the diffusion MRI data.

A structural connectivity matrix was created by transforming the segmentation labels into diffusion space using the registered T1 image. Streamlines that did not start and end in gray matter areas were disregarded. All other streamlines were grouped by their endpoints. For exemplary connectivity matrices with and without the FWM, as well as corresponding streamlines, see Fig 3.

### 2.6 Structural brain network analysis

For in-vivo data of the human brain, no groundtruth structural connectivity matrix (CM) was available, neither for healthy subjects, nor for brain tumor patients. Therefore, we had to rely on other, indirect measures to quantify changes to the CM. We used established metrics for structural CMs, including raw fiber count between regions, inter-subject coefficient of variation, the clustering coefficient as well as intra- and inter-subject correlation. These metrics were partly inspired by metrics used in [34] as well as in [35].

For all metrics, we tested the difference with and without the FWM independently for brain tumor patients and healthy subjects using the paired sample t-test. Regarding difference between patients and controls, the independent sample t-test was utilized.

#### Fiber count

As the most basic metric, the raw fiber count *N* was defined as the total number of fibers included in the connectome. Thus, it includes all fibers connecting distinct GM regions. All fibers that did not end or start in WM, CSF or outside the brain were excluded.

#### Average tract length

The average tract length measures the mean length of all streamlines considered in the CM. It can be expressed as

$$\begin{array}{c} \bar{L}=\frac{1}{N}\sum^{N}L_{i}, \end{array}$$ (1)

where *L*_*i*_ denotes the length in mm of a single diffusion streamline, and *N* the fiber count.

#### Coefficient of Variation

The coefficient of variation (CoV) was proposed by [35] for reproducibility analysis of structural connectomes. For a single entry in the CM, it measures the variability over different acquisitions or different subjects, and can be expressed as

$$\begin{array}{c} CoV_{E}=\frac{\theta_{E}}{\mu_{E}}, \end{array}$$ (2)

with *μ*_*E*_ the mean, and *θ*_*E*_ the standard variation of a specific entry in the CM.

Here, the inter-subject CoV of the total connection strength, i.e. the number of found fiber connections between two regions, was used. Finally, the CoV was averaged over all entries with at least one non-zero count in the whole dataset. In the original paper, CoVs of 0.58 to 0.7 were found for deterministic tractography, depending on the seeding mechanism used. A lower CoV indicates that the CMs in the cohort are more similar, while a higher CoV points to more dissimilar CMs.

#### Binary graph properties

In order to obtain a binarized graph, the connectivity of two regions was set to one when the overall streamline count connecting these regions was higher than a threshold, and set to zero otherwise. While all information about connection strength and distance are discarded in such a binary graph, valuable insights about the topology can be gained.

Two different properties of the binarized graph were computed, the density as well as the clustering coefficient. The density D is obtained by dividing the number of considered connections by the number of all possible connections,

$$\begin{array}{c} D=\frac{2\sum d_{ij}}{n(n-1)}, \end{array}$$ (3)

where i,j = 1,…n, and *d*_*ij*_ denotes whether a connection between regions i and j was found. If D is close to one, the matrix is called dense, while it is considered sparse for a small value of D.

The clustering coefficient C measures the “cliquishness”, i.e., the fraction of neighbors of a node that are directly connected [36]. It is defined as the ratio of all closed triples—a set of three completely connected nodes—to the number of all triples in the graph. Together with the mean path length of the CM, it is often used to quantify “small-worldness” of network architectures [34].

#### Inter-subject correlation

By calculating the Pearson correlation coefficient

$$\begin{array}{c} \rho_{M_{1},M_{2}}=\frac{cov(M_{1},M_{2})}{\sigma_{M_{1}}\sigma_{M_{2}}} \end{array}$$ (4)

between two matrices *M*_1_, *M*_2_, it is possible to calculate the mean correlation for a group of subjects with

$$\begin{array}{c} \rho_{m}=\frac{2}{m(m-1)}\sum_{i}\sum_{j>i}\rho_{ij}, \end{array}$$ (5)

where *i*, *j* = 1, …*m*, *m* refering to the number of subjects in the cohort. With *ρ*_*m*_ = 1, all CMs would be identical (with at least one non-zero entry), while completely random graphs would result in *ρ*_*m*_ = 0.

#### Brain hemisphere correlation

While specific neural functions tend to be lateralized to one side of the brain, the structure of WM in healthy subjects is similar in both hemispheres—the two sides are enantiomorphically related. Morphometric variance between both hemispheres have been shown to be smaller than the differences between subjects [37, 38], and dMRI tractography inter-hemispheric variance seems to be about the same as inter-subject variance [20] in healthy subjects.

We express the left-right similarity as the correlation of the left- and right cortical sub-connectomes. These inter-hemisphere sub-connectomes can be extracted by considering the submatrix from label 3 to 36, and from label 46 to 79. For labels and the respective regions, see S2 Table in the appendix.

Our hypothesis was that an increase in the inter-hemisphere correlation in non-lesioned brains pinpoints a less noisy tractogram. Meanwhile, a stronger increase in this correlation for brain tumor patients is an indicator that the novel fibers found have a corresponding fiber in the other hemisphere, suggesting that the found fibers were indeed true positive connections.

## 3 Results

### 3.1 Total number of fibers

Fiber tracking including the FWM correction resulted in a significant increase in the total number of streamlines that connect two regions, regardless of the cohort, the tracking algorithm, and the seeding mechanism. The relative increase is displayed independently for high grade gliomas (WHO grades III and IV), low grades gliomas (WHO grades I and II) and the control group in Table 1. Independent of the group and tracking methodology, the increase is strongly significant (p<0.0001). However, when using probabilistic tracking, the increase is more pronounced. Comparing glioma patients to healthy controls, the increase in number of fiber tracks is significantly greater for high grade gliomas, but not significantly greater for low grade gliomas.

**Table 1:** Median relative increase of the number of fibers tracked for patients with cerebral gliomas by WHO grade as well as for the healthy controls. Det: deterministic tractography, Prob: probabilistic tractography, WM: white matter seeding, BD: boundary seeding. Significantly (p<0.05) greater increases in patients than in controls are marked in bold. All increases versus tracking without the free water model were strongly significant with p<0.0001.

	Grade III/IV	Grade I/II	Controls
Det/BD	**10.4%**	7.2%	6.3%
Det/WM	**10.9%**	7.8%	5.8%
Prob/BD	**15.0%**	11.0%	9.4%
Prob/WM	**16.2%**	11.0%	9.1%

**Fig 4:**
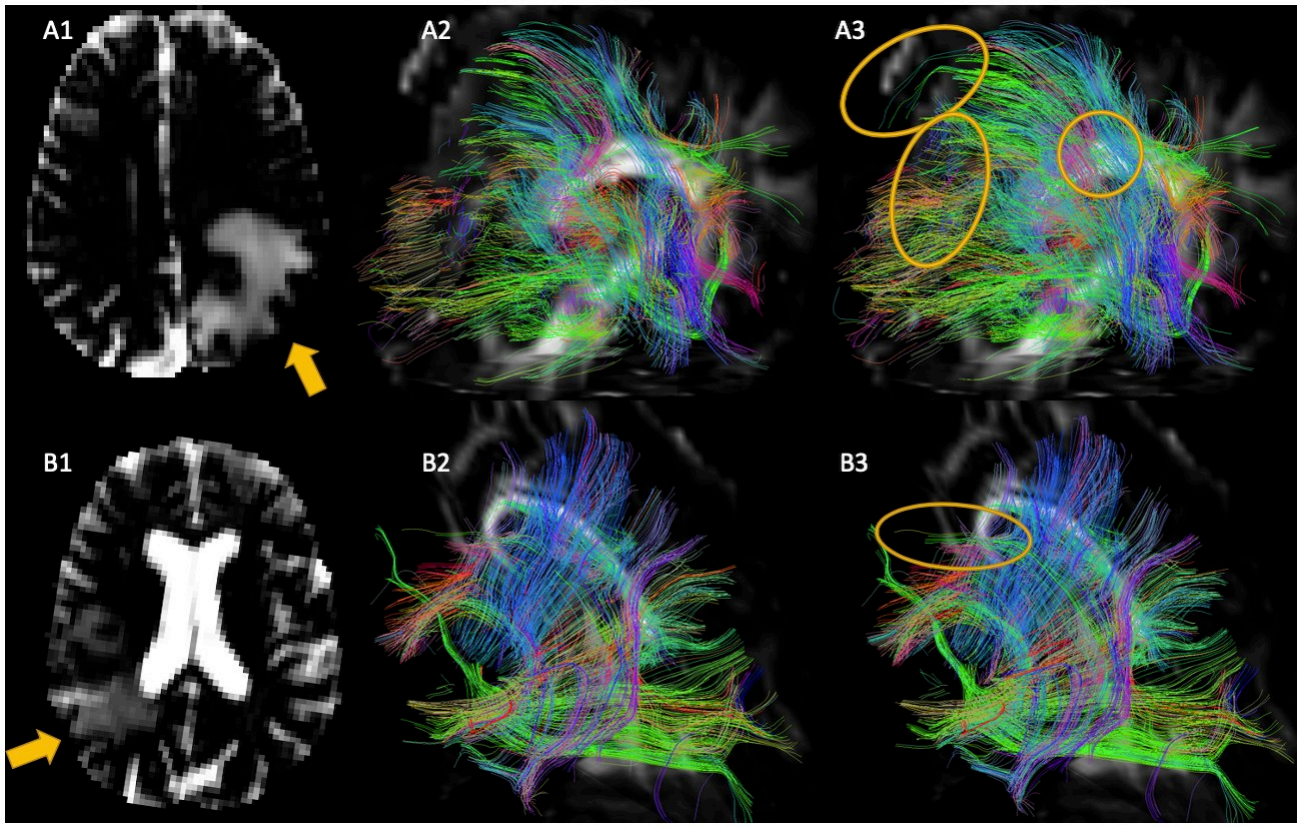
Free-water maps (A1, B1) and boundary-seeded deterministic tractography results for two patients before (A2, B2) and after including free-water effects (A3, B3). In the free-water maps, black indicates a water content of 0, and white one of 100%. The yellow arrows on the free-water maps indicate the direction of view for the tractography visualization, while the yellow ovals highlight changes in tractography. For the tractography visualizations, only one seed point per voxel was utilized, and only the streamlines that pass through the tumormask including edema and infiltrated parts of the brain are displayed. In the upper row (row A), the effects on patient data containing a large edema are visualized, whereas the edema of the patient in the lower row (row B) is less pronounced.

In general, the increase in number of fibers was dependent on the individual anatomy. In Fig 4, the effects on one patient with a large and on a patient with a smaller edema are visualized. Stronger effects can be seen on the patient with the larger edema. For a visualization of the tractography results on a patient with a very large edema, see also Fig 3.

### 3.2 Average tract length

Regarding the average tract length, i.e. the mean distance of all connections in the CM, there was no significant change for all evaluated tracking variations. An overall slight tendency towards longer streamlines could be observed, but single combinations of tracking, seeding, and cohort also led to a slight, non-significant decrease in average length. For the complete results, refer to the appendix (S1 Table).

### 3.3 Coefficient of variation

**Fig 5:**
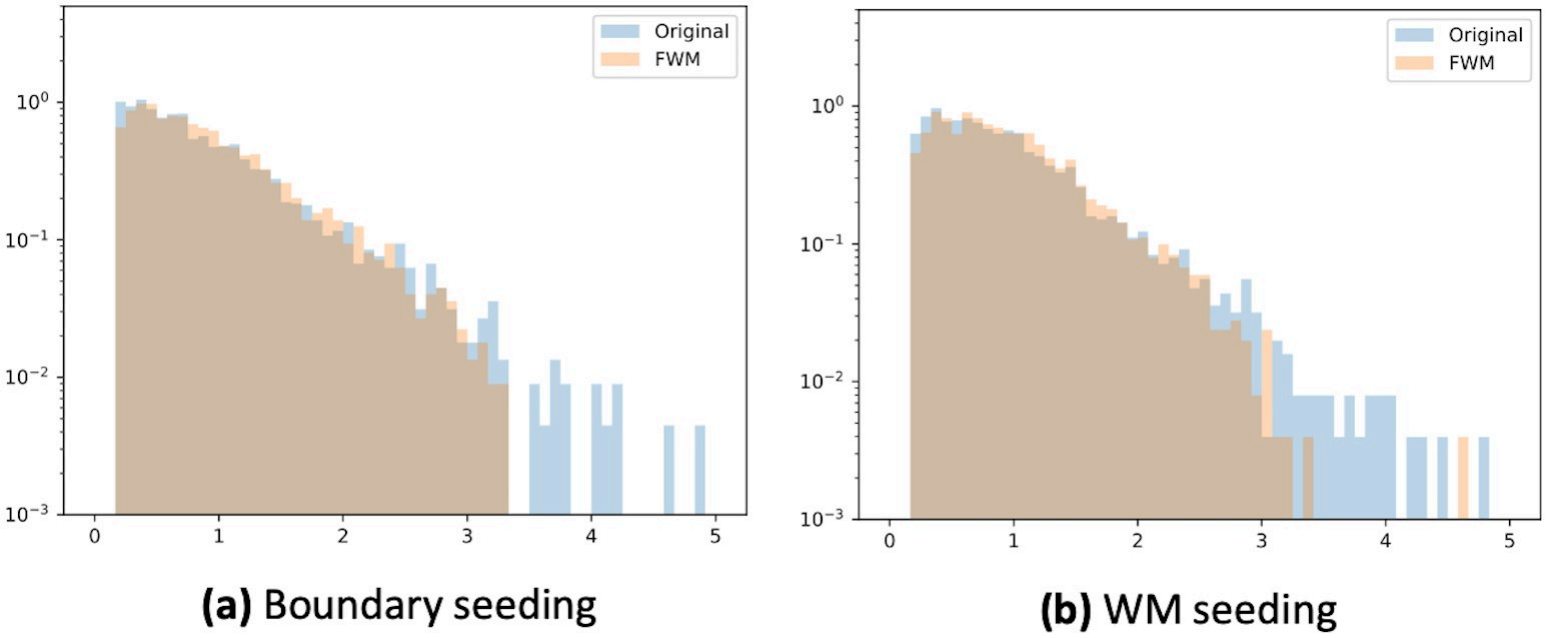
Normalized distribution of the inter-subject coefficient of variation (CoV) for probabilistic tractography in healthy controls. Note the logarithmic scaling of the y-axis. The density of occurrence falls exponentially with the value of the CoV. While the mean of the distribution with- and without the free water model for white matter seeding did not significantly change, after free water modeling, the distribution fell more drastically for higher values of the CoV.

The inter-subject CoV of the mean total connection strength increased significantly in most combinations. Only for probabilistic tractography with WM seeding in the healthy control group, no significant changes could be observed (Table 2). However, for probabilistic tractography in healthy subjects, the distribution of the values was altered, as can be seen in Fig 5. These CoV distributions differed mostly above a certain threshold—the fiber tracks with a CoV of several standard deviations away from the mean seemed to be suppressed when using the FWM. For all other combinations of tracking, seeding, and cohort, the distributions with and without the FWM looked similar, exhibiting approximately exponential decays and reaching zero at about three.

**Table 2:** Coefficient of variation for tumor patients and controls. Det: deterministic tractography, Prob: probabilistic tractography, WM: white matter seeding, BD: boundary seeding, Pat: tumor patients, Ctrls: control group. All increases that were statistically significant are marked in bold (**p<0.05**).

	Pat	FWM Pat	Ctrls	FWM Ctrls
Det/BD	0.463	**0.479**	0.524	**0.543**
Det/WM	0.423	**0.437**	0.503	**0.520**
Prob/BD	0.729	**0.771**	0.910	**0.920**
Prob/WM	0.751	**0.788**	0.950	0.956

### 3.4 Intra-subject similarity of hemispheres

Except for a single case, the deterministic tractography using WM seeding in the patient cohort, including the FWM resulted in a significant increase in the correlation of the individual hemispheres. The correlation with and without the FWM is reported in Table 3. While the similarity of hemispheres was generally more pronounced in the control group, the increase in similarity was stronger for the patients, where the mean increase was 19.8%, compared to the healthy controls where the mean increase was 12.9%.

**Table 3:** Correlation of left- and right brain hemisphere with and without the free water model (FWM). Det: deterministic tractography, Prob: probabilistic tractography, WM: white matter seeding, BD: boundary seeding, Pat: tumor patients, Ctrls: control group. All statistically significant increases are marked in bold (p<0.05).

	Pat	FWM Pat	Ctrls	FWM Ctrls
Det/BD	0.604	**0.615**	0.664	**0.672**
Det/WM	0.569	0.575	0.638	**0.645**
Prob/BD	0.828	**0.850**	0.889	**0.901**
Prob/WM	0.809	**0.830**	0.880	**0.893**

### 3.5 Inter-subject, similarity of cohort

The inter-subject similarity of the cohort, measured as the mean correlation of the CM of each individual subject with all other subjects in that cohort, was significantly increased for most combinations (see Table 4). Similar to the intra-subject similarity of hemispheres, it was more pronounced in the patient cohort with a mean increase of 1.12% compared to the control group (increase of 0.06%).

**Table 4:** Mean inter-subject correlation with and without the FWM of all subjects in the specific cohort. Det: deterministic tractography, Prob: probabilistic tractography, WM: white matter seeding, BD: boundary seeding, Pat: tumor patients, Ctrls: control group. All statistically significant changes are marked in bold (**p<0.05**).

	Pat	FWM Pat	Ctrls	FWM Ctrls
Det/BD	0.526	**0.537**	0.620	**0.627**
Det/WM	0.503	**0.506**	0.628	**0.630**
Prob/BD	0.742	**0.754**	0.840	0.839
Prob/WM	0.711	0.713	0.826	**0.820**

### 3.6 Binary graph metrics

**Fig 6:**
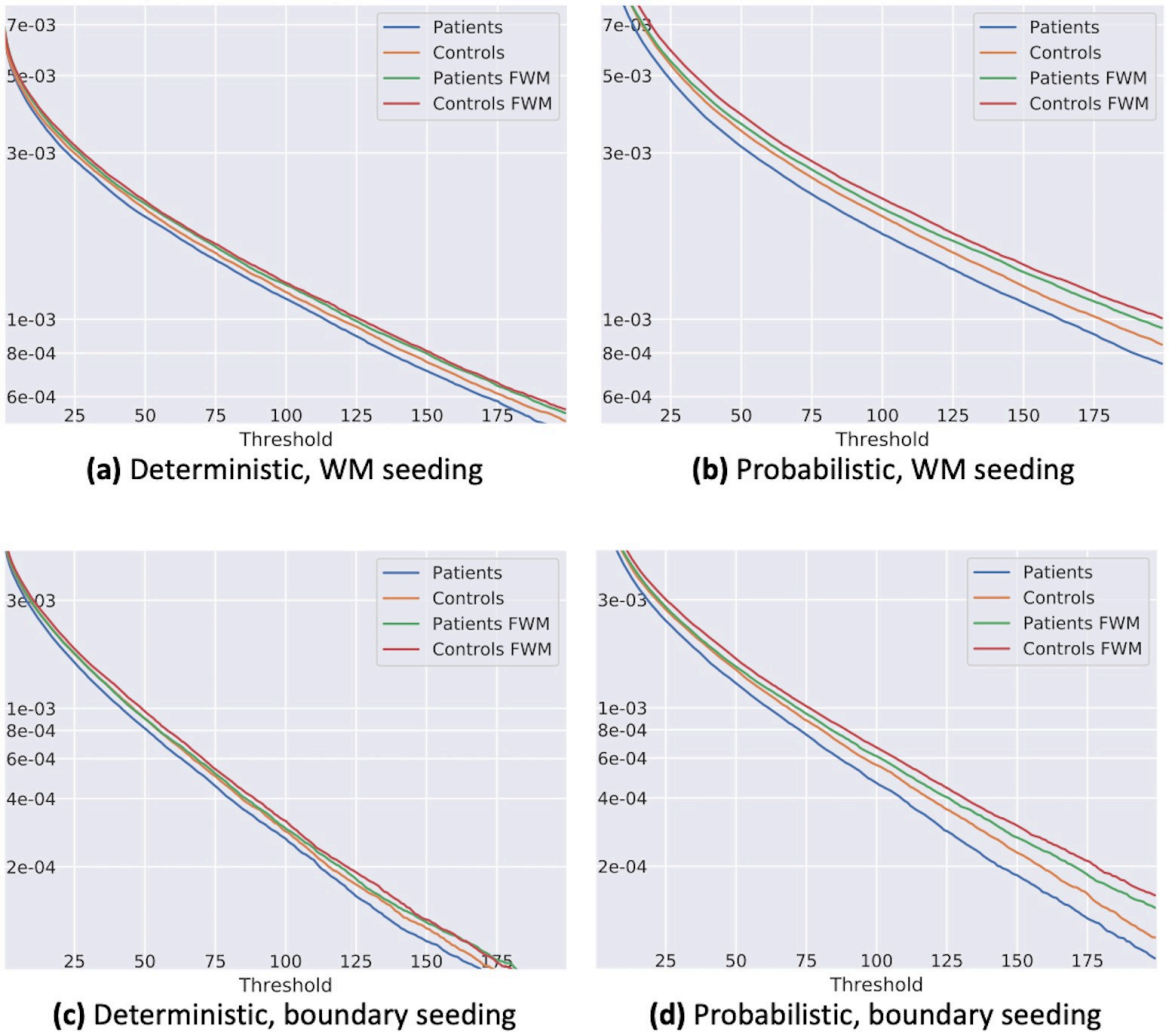
Density of binarized connectivity matrix dependent on the binarization threshold on logarithmic scales. Combinations of the different seeding techniques and tractography methods are displayed in distinct graphs. The absolute density values were strongly dependent on the seeding and tracking algorithm. It can be seen that all functions were heavy-tailed, i.e., while the majority of regions were only connected by a few streamlines, the number of connected tracts with more connections than the threshold fell slower than an exponential function. The number of found tracts was higher in healthy subjects, compared to the brain tumor patients, independent of the inclusion of the free water model (FWM). When including the FWM, the number of tracts found was always higher compared to the direct approach. However, the difference between brain tumor patients and healthy subjects was smaller with the FWM.

The densities of the binarized graphs are plotted as a function of the binarization threshold in Fig 6. The density of CM matrices with FWM was higher than without, and the density of CM matrices in brain tumor patients was lower than in healthy subjects. The difference between healthy subjects and tumor patients was smaller when using the FWM.

Clustering coefficients also increased when using the FWM. For probabilistic tractography, this increase was statistically significant (p<0.05) for the brain tumor cohort as well as for the control group. Including the FWM did not significantly alter clustering coefficients when using the deterministic tracking algorithm in healthy subjects. For brain tumor patients, changes in the clustering coefficient are less strong than when using probabilistic tractography, but were still significant (see Table 5).

**Table 5:** Clustering coefficients of the different CMs. Det: deterministic tractography, Prob: probabilistic tractography, WM: white matter seeding, BD: boundary seeding, Pat: tumor patients, Ctrls: control group. When the FWM clustering coefficient significantly deviated (p<0.05) from the original coefficient, the FWM derived coefficients are marked in bold.

	Pat	FWM Pat	Ctrls	FWM Ctrls
Det/BD	0.522	**0.528**	0.521	0.522
Det/WM	0.552	**0.556**	0.543	0.543
Prob/BD	0.649	**0.665**	0.669	**0.676**
Prob/WM	0.720	**0.736**	0.742	**0.754**

In general, brain tumor patients and healthy controls were more similar when using the FWM. For deterministic tracking, the differences between the groups was not significant, independent of the application of the FWM. However, for the probabilistic tracking, application of the FWM resulted in lower significance or insignificant differences between patients and healthy controls. For probabilistic tractography with WM seeding, the p-value changed from 0.001 to 0.02, for probabilistic tractography with boundary seeding from 0.0006 to 0.05.

## 4 Discussion

We have analyzed how our FWM model influences structural connectivity estimates in brain tumor patients as well as in healthy subjects. As different tractography and seeding techniques strongly influence the results of CMs, the results were analyzed for both deterministic as well as probabilistic tractography, and both WM as well as WM-GM interface seeding approaches.

While the total number of tracked fibers significantly increased when using the FWM, the average tract length did not change significantly. This effect was independent of the seeding technique and whether tractography was performed probabilistically or deterministically. First, this leads to the conclusion that the FWM does not contribute to an overrepresentation of long or short fibers. Second, as the increase in tracked fibers was more pronounced for high grade gliomas than for low grade gliomas or healthy controls, this shows that two different effects, one due to biology, the other one due to limited MRI resolution, can be counteracted with the FWM: For partial volume effects at the border of CSF and the white matter tract, a clear boundary is physically existent, but cannot be exactly measured due to the limited voxel size. Thus, the diffusion signal is averaged from the two juxtaposed volumes. In contrast, in areas infiltrated by vasogenic edema, the edema and the WM tract are not spatially distinguishable on a macroscopic level; a higher resolution would not lead to an improvement. Thus, the uncorrected MRI signal from these regions is not directly suitable for quantification of the fiber tracts. FWM improved peritumoral tractography especially in tumors with substantial perifocal edema, in which conventional tracking algorithms often fail.

With the inter-subject CoV and the correlation coefficients, effects on individual fiber tracts were analyzed. The CoV as well as the inter- and intra-subject correlations increased. In other words, subjects showed higher variance on the level of single fiber tracts but were more similar on the macroscopic connectome level. The increase in connectome level similarity was more pronounced in the patient cohort than in the control cohort. This observation corresponds to our initial hypothesis: Part of the variations in estimated connectivity in tumor patients is not due to true connectivity alterations, but instead a measurement artifact, as tractography based on diffusion MRI is susceptible to free-water effects in presence of peritumoral edema.

The effects of the FWM on the CoV depended on the tractography method: For deterministic tractography, the change of the CoV, while statistically significant, was very small, and effects were comparable between patient and control group. For probabilistic tractography in healthy controls, strongly unusual tracts were suppressed (Fig 5),which can be explained by the necessary fiber direction sampling: The fiber ODF of a voxel containing CSF as well as WM is much wider than the ODF of a voxel containing the same fiber, but no CSF. As the peak of the distribution does not change, deterministic tractography is not much affected, but the probabilistic tractography will produce false positives with a higher frequency. Thus, the reduction of tracts several standard deviations away from the mean is a sign of noise reduction. Meanwhile, the observed increase of the single-tract CoV using probabilistic tractography in patients further indicates the potential of the FWM to improve differentiation of white matter microheterogeneity by demasking free water partial volume effects obscuring true white matter alterations in edema, which is further supported by recent findings [39].

With the metrics derived from the binarized graph, we further analyzed the group differences of brain tumor patients and healthy subjects. Both the difference in density of the matrices as well as the clustering coefficients were less pronounced when including the FWM. Depending on the metric, the difference even dropped from “significant” before including the FWM to insignificant when free water diffusion effects were considered. This decrease of differences is mirrored in the number of fibers and correlation coefficients. For these metrics, the brain tumor patients generally showed lower values compared to healthy subjects, but stronger increases when including the FWM. Thus, after FWM, differences between tumor patients and controls were still persistent. While the differences caused by free-water effects were removed, the differences due to local variations in anatomy were preserved. Hence, when naively comparing tractography results from brain tumor patients to healthy subjects, the influence of the tumor on structural brain connectivity may be overestimated.

A recent study [3] already showed that adding a FWM to tractography improved tracking of a single nerve tract, the arcuate fasciculus, in brain tumor patients. Our results now demonstrate that a FWM also improves whole brain tractography, which we quantify with structural connectivity estimates. Thus, future studies that analyze fiber tracts or structural connectivity in presence of edema should consider the effect of free water on tractography.

Nevertheless, this study has several limitations: Human WM connectivity as measured with diffusion connectomes is subject to variations due to the image acquisition protocol, the employed atlas [34] as well as the utilized tractography method. It would be impossible to evaluate the effects of the FWM with respect to all possible combinations. For example, when using dMRI scans with higher resolutions partial volume effects at the border between CSF and WM decrease naturally. Also, in this study, patients were scanned with a single-shell diffusion acquisition setting. With multi-shell settings, a more accurate determination of the free water fraction would be possible, and thus a more reliable correction for tractography. However, due to time-constraints, multi-shell acquisitions are not always feasible in clinical settings.

Furthermore, we modeled the diffusivity of edema to be equal to the diffusivity of CSF, and the free water content of healthy single fiber WM voxels as zero. While this may be close to reality, it is not 100% exact. Especially in multi-shell acquisitions, a possibility to overcome this limitation would be to not assume fixed isotroptic diffusivities, but instead to model CSF, GM, and edema with isotropic diffusivity spectra [40]. Still, in our previous publication [19], we have shown that our current approach seems to be more or at least as accurate for determining relative water content and resulting FA values as comparable approaches [13, 18, 41] on single-shell and multi-shell data. Moreover, comparing our method to previously published approaches, our approach provides more conservative results regarding the predicted water compartment. While it predicts a water fraction between 0.01 and 0.02 for healthy white matter, findings of 0.1±0.13 in healthy brain tissue of tumor patients were reported using a spatial regularization based free water correction [17]. In consequence, most other FWM approaches will presumably show even stronger effects on whole brain tractography. Therefore, as it is still unclear how large the absolute modeling error of our and comparable approaches is, a more conservative approach is well suited to estimate free water effects on tractography.

Nevertheless, a quantitative evaluation of the modeling error of our FWM method as well as other FWM methods will be necessary in the future. A quantification of the model error would be possible using dedicated MRI acquisition protocols and diffusion phantoms, such as presented by [42]. Such an acquisition is not possible in standard clinical settings, where the acquisition time remains a crucial factor. However, it can be used to quantify the accuracy of our FWM, as well as the accuracy of other FWM approaches that aim at solving this ill-posed problem that arises in clinical settings.

## 5 Conclusion

We analyzed the effects of deep learning free water mapping and elimination based on individually extracted data on structural connectivity analysis of brain tumor patients and healthy subjects. Independent of the tractography algorithm and seeding method, the FWM leads to more, but not to longer tracked fibers. Differences in connectivity estimates between brain tumor patients and healthy controls generally decreased when including the FWM. Thus, we hypothesize that—especially in high grade glioma—the FWM improves peritumoral tractography, which might be relevant for presurgical planning and postoperative outcome. Further, estimates of structural connectivity impairments in brain tumor patients appear to be exaggerated when effects of edematous regions on tractography are not considered. Future studies should consider this effect when comparing diffusion MRI derived measurements from lesioned and healthy control brains.

## Supporting information

S1 Table Average tract length for brain tumor patients and healthy controls. (PDF)

S2 Table Parcellation labels, cortical and subcortical regions of interest. (PDF)

S1 File Free-water maps and matrices of patients and controls. All utilized connectivity and length matrices as comma-separated csv files, and the free-water maps as nifti files.
(ZIP)
