# Supplementary material for: Analyzing the effects of free water modeling by deep learning on diffusion MRI structural connectivity estimates in glioma patients
Source: PLoS One. 2020 Sep 25;15(9):e0239475. doi: 10.1371/journal.pone.0239475 (PMC7518620; doi:10.1371/journal.pone.0239475)
Supplement: S1 Table — (PDF) [file pone.0239475.s001.pdf]

Appendix S1 of: Analyzing the effects of free water modeling by deep learning on diffusion MRI structural connectivity estimates in glioma patients

Leon Weninger[1], Chuh-Hyoun Na[2], Kerstin Juetten[2], Dorit Merhof[1],

1 Imaging & Computer Vision, RWTH Aachen University, 52074 Aachen, Germany
2 Department of Neurosurgery, University Hospital RWTH Aachen, 52074 Aachen, Germany

S1 **Table.** Average tract length for brain tumor patients and healthy controls. Det: deterministic tractography, Prob: probabilistic tractography, WM: white matter seeding, BD: boundary seeding, Pat: tumor patients, Ctrls: control group. No changes were statistically significant ($p<0.05$).

	Pat	FWM Pat	Ctrls	FWM Ctrls
Det/BD	53.0	52.8	57.3	57.5
Det/WM	58.1	58.2	62.3	62.4
Prob/BD	79.9	80.6	87.1	87.4
Prob/WM	92.1	92.6	98.6	99.1
