# Supplementary material for: Analyzing the effects of free water modeling by deep learning on diffusion MRI structural connectivity estimates in glioma patients
Source: PLoS One. 2020 Sep 25;15(9):e0239475. doi: 10.1371/journal.pone.0239475 (PMC7518620; doi:10.1371/journal.pone.0239475)
Supplement: S2 Table — (PDF) [file pone.0239475.s002.pdf]

Appendix S2 of: Analyzing the effects of free water modeling by deep learning on diffusion MRI structural connectivity estimates in glioma patients

Leon Weninger[1], Chuh-Hyoun Na[2], Kerstin Juetten[2], Dorit Merhof[1],

1 Imaging & Computer Vision, RWTH Aachen University, 52074 Aachen, Germany
2 Department of Neurosurgery, University Hospital RWTH Aachen, 52074 Aachen, Germany

S2 **Table.** Parcellation labels: Cortical and subcortical regions of interest

Label	Name	Label	Name
3	Left frontalpole cortex	46	Right frontalpole cortex
4	Left medialorbitofrontal cortex	47	Right medialorbitofrontal cortex
5	Left lateralorbitofrontal cortex	48	Right lateralorbitofrontal cortex
6	Left parsorbitalis cortex	49	Right parsorbitalis cortex
7	Left parstriangularis cortex	50	Right parstriangularis cortex
8	Left parsopercularis cortex	51	Right parsopercularis cortex
9	Left rostralmiddlefrontal cortex	52	Right rostralmiddlefrontal cortex
10	Left caudalmiddlefrontal cortex	53	Right caudalmiddlefrontal cortex
11	Left superiorfrontal cortex	54	Right superiorfrontal cortex
12	Left precentral cortex	55	Right precentral cortex
13	Left paracentral cortex	56	Right paracentral cortex
14	Left insula cortex	57	Right insula cortex
15	Left rostralanteriorcingulate cortex	58	Right rostralanteriorcingulate cortex
16	Left caudalanteriorcingulate cortex	59	Right caudalanteriorcingulate cortex
17	Left posteriorcingulate cortex	60	Right posteriorcingulate cortex
18	Left isthmuscingulate cortex	61	Right isthmuscingulate cortex
19	Left entorhinal cortex	62	Right entorhinal cortex
20	Left fusiform cortex	63	Right fusiform cortex
21	Left parahippocampal cortex	64	Right parahippocampal cortex
22	Left inferiortemporal cortex	65	Right inferiortemporal cortex
23	Left temporalpole cortex	66	Right temporalpole cortex
24	Left middletemporal cortex	67	Right middletemporal cortex
25	Left superiortemporal cortex	68	Right superiortemporal cortex
26	Left transversetemporal cortex	69	Right transversetemporal cortex
27	Left bankssts cortex	70	Right bankssts cortex
28	Left postcentral cortex	71	Right postcentral cortex
29	Left supramarginal cortex	72	Right supramarginal cortex
30	Left inferiorparietal cortex	73	Right inferiorparietal cortex
31	Left superiorparietal cortex	74	Right superiorparietal cortex
32	Left precuneus cortex	75	Right precuneus cortex
33	Left cuneus cortex	76	Right cuneus cortex
34	Left lateraloccipital cortex	77	Right lateraloccipital cortex
35	Left pericalcarine cortex	78	Right pericalcarine cortex
36	Left lingual cortex	79	Right lingual cortex
37	Left caudate	80	Right caudate
38	Left putamen	81	Right putamen
39	Left pallidum	82	Right pallidum
40	Left thalamus	83	Right thalamus
41	Left thalamus proper	84	Right thalamus proper
42	Left hippocampus	85	Right hippocampus
43	Left amygdala	86	Right amygdala
44	Left accumbens area	87	Right accumbens-area
45	Left ventralDC	88	Right ventralDC
